# Clinical Notes

**Published:** 1888-02

**Authors:** Clarence Willard Butler


### CLINICAL NOTES.

### Clarence Willard Butler, M. D.

Nausea of Pregnancy.—Mrs. C. B., age thirty-six, at the third month of her fourth pregnancy, suffers from continuous and persistent nausea without vomiting. Ip., Sep., etc., have not relieved. Her mental symptoms, which had been concealed from me heretofore, were as follows: great *fear;* fears she will not survive her confinement; anxious and timorous; does not dare go on to the street alone; in constant fear when in crowded places, as church, market, etc., but does not know *what* she fears. Aconite$^{45m}$, F., one dose, relieved promptly not only the nervous apprehension but the nausea as well. My friend J. H. Wilson, M. D., of Bellefontaine, Ohio, guided by the above mental symptoms, some years ago cured a case of "morning sickness" brilliantly with Acon. in a low potency.

Mrs. F. W. A., age twenty-nine, at the fourth month of her second pregnancy, complained of severe nausea and occasional vomiting, which annoyed her only late in the afternoon and evening; appetite good when not nauseated; no thirst; tendency to be chilly all the time (in August). Puls.$^{mm}$ (Tafel), one dose. Entire and prompt relief.

Sciatica.—Mr. H. M. R., age forty-eight, carpenter by trade, has been working during the cold weather in an exposed place. For three days has suffered from a severe drawing pain in the left leg, commencing at the nates and extending down the posterior aspect of the thigh to the popliteal space and on to the calf of the leg; better while lying quietly, with the leg extended, and while walking slowly, but is *unbearable while sitting.* Rhus tox. and Pulsatilla afforded no relief (of course not), but for the two days he was wasting with these drugs he grew steadily worse till the only relief he had was by the recumbent posture. Amm. mur.$^{cm}$ (H. S.) relieved in two hours and cured in a day and a half. He received but one dose.

Pruritus Ani.—Mr. A. B., Jr., age thirty, a periodical dipsomaniac, tall, slender, of light complexion, and nervous temperament, has been for the last four years a sufferer from this disease. It troubles him all the time, but is much worse at night. The itching in and about the anus and forward along the perineum is so severe at night as to deprive him of sleep. It is relieved *while* scratching vigorously, and by application of cold water. When relieved by the compress he drops off to sleep, it quickly wakens him, and it is not unusual with him to be obliged to rise to apply these compresses every twenty or thirty minutes all night long. He has lost flesh and strength, has little appetite, and is very nervous and irritable. After several drugs had been exhibited without benefit, Amm. c.$^{cc}$ (Dunham), in repeated doses for forty-eight hours, cured him in ten days. For more than seven months to the present time there has been no return of the disease.

Dysmenorrhœa.—Miss L. S., age twenty-five, a school teacher, of large frame, coarse fibre, dark complexion, with tendency to adipose, has suffered for three years with dysmenorrhœa. Menses are regular as to time, rather profuse, natural in color, somewhat inclined to be offensive in odor. Leucorrhœa in inter-menstrual period. For the first twelve or eighteen hours of menstruation she has severe cramping pains low in the abdomen, accompanied with nausea, vomiting, and diarrhœa. The bowels move three or four times, usually loose and profuse (she is habitually constipated), but the vomiting continues throughout the first day. Amm. c.$^{cm}$ (H. S.), one dose. Two weeks after taking the Amm. c. menstruation appeared at the regular time. One stool at the commencement, freer than usual, but not diarrhœa; slight nausea but no vomiting. She taught all this first day of menstruation, which she had not been able to do before for many months. Six weeks later menstruation normal and painless; no nausea, no vomiting; no further trouble to this time, now more than a year. No report regarding the leucorrhœa was made. She received but one dose of the remedy.

Heart Disease—Palliation.—Mrs. S., age about sixty-five, has had a mitral insufficiency for several years. For the last three months has been confined to her room, and her condition is now pitiable in the extreme. She is unable to lie down, her legs and left arm are enormously swollen, and the abdomen filled with dropsical effusion. The skin of the legs below the knee is dark red, tense, and glistening, while upon the left calf a large ulcer marks the death of tissue from the interference with circulation. A thin, excoriating fluid oozes continually from the skin of the legs and causes a sensation of burning in the skin. Under medication she has been relieved of her suffering very largely, so that now she complains of no *pain* except the burning already mentioned. But she is nervous, *restless*, sensitive to noises of all kinds, and sleepless. A feeling of excessive "nervousness" causes the restlessness and the sleeplessness, she says, but cannot explain more definitely except that it makes her unbearably miserable. *Beseeching mood.* "Oh! dear doctor, pray *do* give me something to relieve me! You must do something; help me, doctor, for I cannot bear this nervousness." Coffea cr.$^{cm}$ (H. S.), one dose, was followed by the most gratifying and ever-surprising relief. The first night after receiving it she slept about six hours continuously, and so quietly that her nurse repeatedly went to her to see if she was living. Several times since she has needed the same remedy, the only one she takes when she evinces a tendency to the nervous state which called for it, and it has never failed to give prompt relief. Her nurse (trained in an allopathic hospital) will not believe that it is not some preparation of Morphine, and wonders that it does not produce the unpleasant features of that drug, which she has so often observed when it has been administered to her former patients.

